# Fully Printed, Wireless, Stretchable Implantable Biosystem toward Batteryless, Real‐Time Monitoring of Cerebral Aneurysm Hemodynamics

**DOI:** 10.1002/advs.201901034

**Published:** 2019-08-07

**Authors:** Robert Herbert, Saswat Mishra, Hyo‐Ryoung Lim, Hyoungsuk Yoo, Woon‐Hong Yeo

**Affiliations:** ^1^ George W. Woodruff School of Mechanical Engineering Institute for Electronics and Nanotechnology Georgia Institute of Technology Atlanta GA 30332 USA; ^2^ Department of Biomedical Engineering Hanyang University Seoul 04763 South Korea; ^3^ Wallace H. Coulter Department of Biomedical Engineering Parker H. Petit Institute for Bioengineering and Biosciences Neural Engineering Center Center for Flexible and Wearable Electronics Advanced Research Institute for Materials Georgia Institute of Technology Atlanta GA 30332 USA

**Keywords:** aerosol jet 3D printing, aerosol nanoparticles, batteryless wireless monitoring, hemodynamics, stretchable hybrid electronics

## Abstract

This study introduces a high‐throughput, large‐scale manufacturing method that uses aerosol jet 3D printing for a fully printed stretchable, wireless electronics. A comprehensive study of nanoink preparation and parameter optimization enables a low‐profile, multilayer printing of a high‐performance, capacitance flow sensor. The core printing process involves direct, microstructured patterning of biocompatible silver nanoparticles and polyimide. The optimized fabrication approach allows for transfer of highly conductive, patterned silver nanoparticle films to a soft elastomeric substrate. Stretchable mechanics modeling and seamless integration with an implantable stent display a highly stretchable and flexible sensor, deployable by a catheter for extremely low‐profile, conformal insertion in a blood vessel. Optimization of a transient, wireless inductive coupling method allows for wireless detection of biomimetic cerebral aneurysm hemodynamics with the maximum readout distance of 6 cm through meat. In vitro demonstrations include wireless monitoring of flow rates (0.05–1 m s^−1^) in highly contoured and narrow human neurovascular models. Collectively, this work shows the potential of the printed biosystem to offer a high throughput, additive manufacturing of stretchable electronics with advances toward batteryless, real‐time wireless monitoring of cerebral aneurysm hemodynamics.

## 1 Introduction

Recently, a number of miniaturized, implantable electronics have been enabled by advances in microfabrication techniques.^1^, ^2^, ^3^ In combination with soft materials, stretchable and flexible devices, including biosensors, with low form factors have been developed. The practical use of these sensors heavily depends on their wireless monitoring capabilities and manufacturing reliability and scalability. Despite this requirement, the development of implantable electronics often lacks functional interrogation distances and relies on slow, low‐throughput fabrication procedures. The next class of implantable electronics will rely on highly stretchable and flexible forms while also offering wireless operation without the use of rigid electrical components. Many current wearable and implantable electronics still rely on rigid circuitry to enable wireless interrogation. These rigid components are incompatible with implantation in soft tissues or blood vessels due to mechanical mismatch and bulky packaging. A variety of implantable devices have been recently developed for vascular applications.^2^, ^3^ Such devices include an inductive stent with a capacitive diaphragm pressure sensor. But, those devices require the use of a bridging wire that is susceptible to fracture, while the readout distance is limited to less than 3 cm in air.^4^ Another work has focused on a multifaceted, wireless biodegradable stent with the use of a number of active circuit components.^5^ Other studies have developed miniaturized sensors for integration with stents or wrapping around the blood vessel, but have readout distances insufficient for practical applications.^6^, ^7^ Currently, an implantable pressure sensor is commercially available, but consists of a bulky, rigid electronics package intended for pressure sensing in larger diameter blood vessels.^8^ To accomplish passive, wireless monitoring, devices must offer excellent electrical properties, in particular highly conductive components. Moreover, rapid protoyping and reliable fabrication procedures are attractive capabilities to advance to the next class of stretchable and implantable electronics.

One of the most challenging implant locations is in the highly contoured and narrow cerebral arteries where cerebral aneurysms exist in about 6% of the population.^9^ Cerebral aneurysms result from weakened sections of blood vessels, and the continuous incoming flow to the ballooned section of the vessel may cause rupture, often resulting in death or permanent damage.^10^, ^11^ With these risks, it is critical to employ effective treatments and follow‐up monitoring. Recently, microstructured flow diverters have shown a 76% complete occlusion rate, greatly reducing the risk of rupture.^12^, ^13^ Although flow diverters indicate high effectiveness, a post‐treatment monitoring method is still required for assessment of cure progress and sac occlusion. In the vascular system, implantable sensors must achieve high stretchabilities and flexibilities, while mantaining an extremely low‐profile form to not disrupt or impede blood flow, particularly in cerebral arteries where vessel diameters are as low as 3 mm in diameter. To improve the postmonitoring capability, our prior works investigated a low‐profile capacitive flow sensor, which still needs wireless sensing capability for a fully implantable system.^14^, ^15^ Widely used wireless interrogation for implantation primarily includes radiofrequency communication and inductive coupling.^1^, ^2^, ^3^ Some recent works with rigid electronics have attempted the integration of the inductive coupling method with medical stents.^6^, ^16^ However, they still show limited readout distances, less than 3 cm in air, but wirelessly transmitted signals attuenate rapidly in tissue. Cerebral aneurysm detection often is required to reach arteries over 5 cm from the skin.^17^ Other devices rely on bulky packaging that disrupts normal hemodynamics.^8^ More importantly, the rigid electronics based on metals and plastics have a huge risk of thrombosis and flow disruption in a highly contoured blood vessel for monitoring of hemodynamics. Recently, aerosol jet printing (AJP) has been introduced to address the issues of conventional fabrication techniques. AJP, compared to conventional processes, allows for faster and reliable fabrication and scalable manufacturing via direct printing, digital designing, and optimized control. However, previous demonstrations of AJP‐based electronics have been limited to passive electrical components, development of transistors, and flexible substrates.^18^, ^19^, ^20^ One study developed a freestanding interconnect for use as a strain sensor and humidity sensor.^21^ However, conductive traces were fabricated as a mixed polyimide (PI) and silver nanoparticle (AgNP) composite that resulted in resistances still too high for use in wireless devices despite an overall thickness near 30 µm. Currently, AJP has not been applied for the development of wireless, stretchable electronics.

Here, this work addresses these issues by introducing a fully passive wireless, low‐profile capacitive sensor and coil inductor with enhanced readout distance via comprehensive understanding and optimization of AJP materials and fabrication methods. To our knowledge, this is the first demonstration of an AJP‐enabled highly stretchable capacitive system for wireless monitoring and first optimization of AJP for thin, multilayer devices. The fully additive process involves high precision printing of four aligned layers followed by seamless integration on a soft elastomer. The extremely low‐profile and stretchable structure allows the device to be conformally integrated onto a medical stent and deployed via conventional catheter procedures. Utilization of a new inductive coupling method to monitor hemodynamics enables a batteryless, wireless detection of the printed sensors at distances surpassing existing devices. Optimization of relevant parameters in the inductive coupling system is performed by analytical and computational calculations. In vitro experimental study demonstrates the performance of the optimal sensor coils in wireless detection of resonant frequencies through air, saline, and meat (beef). The sensor is demonstrated in two highly contoured biomimetic cerebral aneurysm models, indicating high sensitivity to incoming aneurysm flow. These results indicate feasibility for possible in vivo applications and advance the application of AJP to high‐performance, stretchable electronics.

## 2 Results and Discussion

### 2.1 Overview of AJP‐Enabled Fabrication

**Figure 1:**
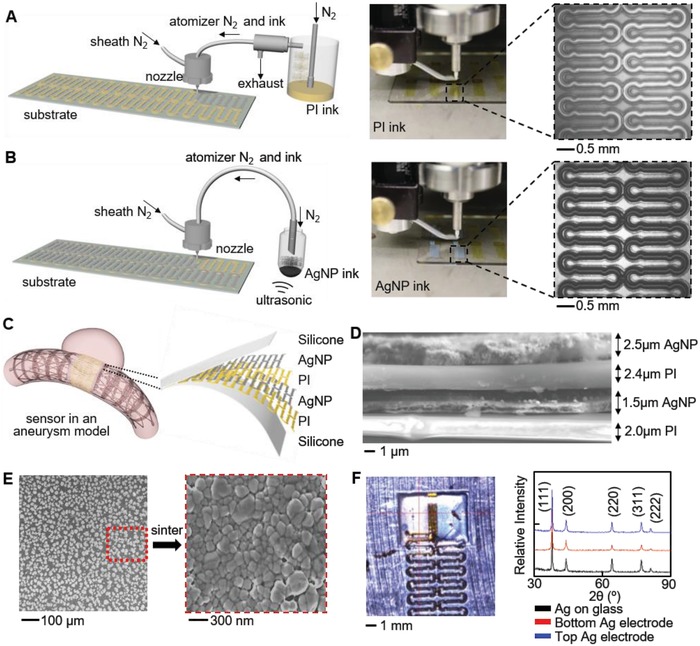
AJP fabrication and material characterization. A) Illustration and image of AJP deposition of PI using a pneumatic atomizer (left). Enlarged view shows a printed PI trace (right). B) Overview of Ag deposition with an ultrasonic atomizer (left) and enlarged view of a patterned Ag on PI (right). C) Illustration of the AJP‐enabled fabrication of an implantable flow sensor in an aneurysm model. Inset shows the multilayered structure of the sensor package. D) Cross‐sectional SEM image displaying multilayered sensor structure. E) SEM images of AgNPs as printed (left) and after a sintering process, showing clusters (right). F) X‐ray diffractometer characterization of a sintered AgNPs on glass slide, bottom Ag electrode on PI, and top Ag electrode on PI. No change in crystalline structure is observed for the three cases.

**Figure** **1**A,B illustrates the overall manufacturing process of a wireless flow sensor (further details in Figure S1, Supporting Information). The printing of the implantable biosensor is performed via the AJP method (Aerosol Jet 200, Optomec) on a precleaned glass slide with spin‐coated polymethyl methacrylate (PMMA). The sensor utilizes polyimide as a bottom supporting layer for a direct integration with a soft elastomer and as the dielectric layer (Figure 1C). The PI ink is composed of a mixture of a precursor (PI‐2545, DuPont) and solvent (1‐methyl‐2‐pyrrolidinone; NMP, Sigma‐Aldrich). The ink is atomized in the pneumatic atomizer and deposited via a 300 µm‐diameter nozzle (Figure 1A). Printing speeds were maintained at 10 mm s^−1^ to maintain small feature sizes and low fabrication times. Movie S1 (Supporting Information) demonstrates the AJP of the PI dielectric layer for a sensor. After curing of the printed PI at 240 °C for 1 h, the surface is plasma treated to improve surface adhesion between PI and the following silver (Ag) layer for a sensor. An AgNP ink (Ag40XL, UT Dots) is mixed with xylene (m‐Xylene, Sigma‐Aldrich) and atomizes in the ultrasonic atomizer before printing with a 200 µm diameter nozzle (Figure 1B). Movie S2 (Supporting Information) shows the printing of the top Ag electrode. The printed AgNPs act as the capacitive electrodes in the parallel‐plate structure shown by the cross‐sectional scanning electron microscope (SEM) image in Figure 1D (further details in Figure S2, Supporting Information). The cross‐section displays the dark layers of PI and bright, conductive layers of AgNP. Due to the nonuniform morphologies of the printed PI and AgNPs, there is variable thickness shown in the image. However, this was not observed to affect mechanical or electrical performance. Sintering the printed AgNPs at 240 °C for 1 h yields a high density of the printed AgNPs as demonstrated by the SEM image (Figure 1E).^22^ X‐ray diffraction study of the two printed silver layers indicates the extra sintering time (2 h) for the bottom AgNP layer does not significantly change its composition (Figure 1F). After the final sintering phase, the sensor is transferred to a thin layer of a silicone elastomer (Ecoflex GEL, Smooth‐On) that is spin‐coated on a polyvinyl alcohol (PVA) film by dissolving PMMA in acetone. Silver paste attaches copper wires to the contact pads prior to a top encapsulation layer (Ecoflex 0030, Smooth‐On). The sensor may be transferred with water soluble tape or with the use of tweezers. A small amount of elastomer connects a point at the top and bottom of the sensor to a stent and then the PVA film is dissolved. This integration process enables the sensor to conform to the stent with high flexibility and stretchability.

### 2.2 Optimization of Multilayer AJP for Capacitive Sensor

**Figure 2:**
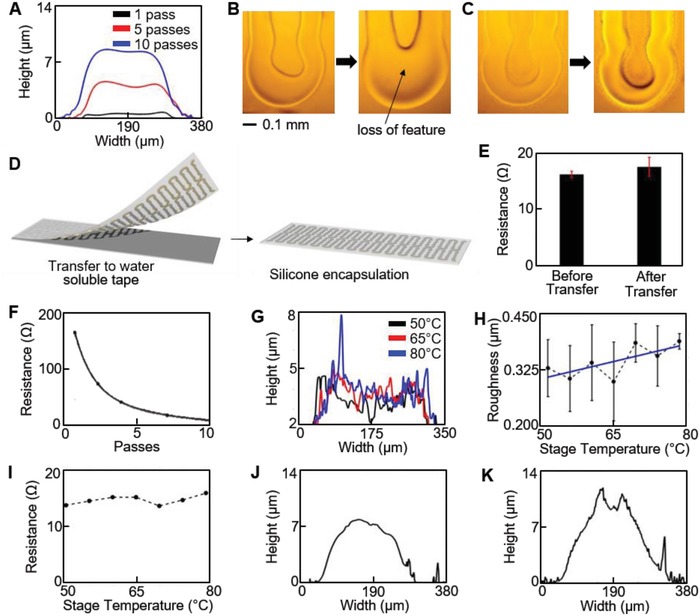
Optimization of AJP processes for a thin, multilayer stretchable biosensor. A) Cross‐section profiles of printed PI layers, indicating a 4:1 ink ratio is optimal to achieve a thicker layer without increasing feature width. B) Microscopic images of 1:1 PI ink with 1 and 5 passes that indicate loss of feature width. C) Microscopic images of 4:1 PI ink from 1 to 5 passes, showing a clear feature. D) Illustration of a transfer printing of a printed material onto a silicone elastomer. E) Comparison of electrical resistance before and after the transfer, showing negligible change of the resistance. F) Decreased electrical resistance with printed multiple passes of AgNP. G,H) Enhanced roughness of the printed AgNP with decreased stage temperatures from 80 to 50 °C. I) Variation of stage temperature from 80 to 50 °C that does not affect resistance of sintered AgNPs. J) Cross‐section profile of dielectric layer printed on bottom PI and AgNP layers indicates a dielectric thickness of ≈2.5 µm. K) Cross‐section profile of completed sensor displays low‐profile form with maximum thickness of 12.4 µm.

To enable high‐throughput manufacturing of mechanically stable sensors, ink and printing parameters are comprehensively studied. For the first time, AJP parameters are optimized for thin, multilayer printing of stretchable electronics. High stretchability is achieved by transferring the sensor from a PMMA‐covered glass slide to silicone elastomer. The quality of printed PI patterns and transfer ability were characterized to identify proper parameters to print the support PI layer. Key parameters include PI to NMP ratio, number of passes, and stage temperature. For successful transfer, the supporting PI layer is thickened to mechanically support the AgNP trace deposited on it. One method to increase the thickness is to thicken the PI ink by decreasing the NMP content. Ratios of PI to NMP include 5:1, 4:1, 3:1, 2:1, and 1:1. The ability to atomize the inks is critical for high quality printing, but the 5:1 ratio is more difficult to maintain consistent printing due to insufficient atomization and nozzle clogging. However, the 4:1 ink printed high quality traces and rapidly thickened with multiple passes without a significant increase in width (**Figure** **2**A). Profiles were measured by a profilometer (Dektak 150). Although the thinner inks can also be used to reach thick traces, more passes are required. The combination of additional passes and thinner ink resulted in spreading during deposition and loss of features, as shown in Figure 2B for the 1:1 ink from 1 pass to 5 passes (further details in Figure S3, Supporting Information). However, the 4:1 ink maintained features without spreading, as shown in Figure 2C for 1 to 5 passes. Stage temperature did not have any significant effect on line width or thickness for the 4:1 ink. The thicker 4:1 PI ink minimizes the fabrication time and maintains high quality features. **Table** **1** lists printing parameters for PI. Transfer tests were performed to determine the minimum number of passes, or thickness, to ensure transfer of AgNP to silicone. The transfer process, illustrated in Figure 2D, involved dissolving the sacrificial PMMA layer and water soluble tape. These tests indicate that 3 passes of 4:1 PI ink are sufficient for transfer. Three passes of the mixed PI ink provided a sufficiently thick (2.7 µm) layer with consistent 248 µm wide features (**Table** **2**). Measuring the minimal resistance change of the AgNPs before and after transfer confirmed success (Figure 2E). The PI layer ensures mechanically stable transfer to elastomer without cracking or deformation (Figure S4, Supporting Information). Although additional NMP enables smaller features via a smaller diameter nozzle, it results in thinner layers unsuitable for a follow‐up transfer onto an elastomer.

**Table 1:** Printing and material parameters

Parameter	PI	Ag
Ink Solvent	NMP	m‐Xylene
Sheath rate [ccm]	20	30
Atomization rate [ccm]	1400 (exhaust) 1470 (atomization)	30
Nozzle diameter [µm]	300	200
Printing speed [mm s^−1^]	10	10
Stage temperature [°C]	80	65
Curing/sintering temperature [°C]	240	240
Modulus [GPa]	1.5 × 10^9^	52 × 10^9^

**Table 2:** Dimension of a sensor, composed of multilayers

Dimension [µm]	PI	Ag	Complete sensor
Thickness	2.7 ± 0.05	4.1 ± 0.4a)	12.4 ± 0.6
Width	248.3 ± 5.7	188.9 ± 20.2	305.9 ± 55.3

a) Skin depth of silver at 6.2 MHz is 25.5 µm.

The second challenge of AJP for thin, multilayer printing is identifying key parameters to control print profiles to prevent shorting between conductive layers. The sensor discussed here uses PI ink as the dielectric layer between two AgNP electrode layers. The previously reported AJP‐enabled structure^21^ relied on a thick layer deposition for preventing shorting between conductive layers, but lowered sensitivity. Optimization of a thin dielectric layer deposition is important, since minimizing the dielectric thickness will increase sensitivity.^23^ Prior to optimization, the two electrode layers were consistently and frequently electrically connected despite depositing PI between the layers. Unlike conventional fabrication techniques, multilayer printing by AJP yields a nonplanar feature that depends on printing parameters and inks.^24^, ^25^ The profile, particularly of printed AgNP, has been extensively studied in terms of nozzle size, print speed, and focusing ratio to achieve high aspect ratios. Aspect ratio, along with other criteria, impacts the ability to prevent sensor shorting, minimize device thickness, and maximize sensitivity. The profile of the bottom AgNP layer can prevent the dielectric layer from fully insulating it from the top AgNP layer. If the Gaussian profile of the bottom AgNP layer is narrow, the dielectric layer may not cover the AgNP peak before drying. For electrical performance, a thick AgNP layer is desired, which further complicates proper coverage. Increasing the thickness by multiple passes reduces resistance along the AgNP path (Figure 2F). Based on previous studies, a low sheath to atomizer flow ratio enables lower aspect ratio.^25^ Here a ratio of 1 is maintained and other parameters are listed in Table 1. In order to enable a lower aspect ratio, xylene content in the commercial AgNP ink was increased to 140% by volume to promote spreading of the AgNPs on the supporting PI layer and allow for wider, flatter traces. A minimal increase in resistance occurs when xylene content was increased from 15% to 150% (Figure S5A, Supporting Information). As shown in Figure 2A, the supporting PI layer forms a concave shape, which contains AgNP ink as it spread and dried. To further slow the ink drying, the effect of stage temperature was varied between 50 and 80 °C. Lowering the stage temperature does not have a significant impact on line width, likely due to the concave PI on which it was printed and the high xylene content, but large peaks of AgNP ink less frequently occurred (Figure S5, Supporting Information). Figure 2G displays three profiles, and a large peak is seen for the maximum stage temperature (80 °C). This was quantified by recording the mean roughness value at the tested temperatures. Figure 2H shows an overall trend of increasing roughness with increasing temperature. Printing complete sensor samples indicated that a temperature below 65 °C significantly reduced the frequency of electrical shorting between AgNP electrodes. Across the temperature range, the after‐sintering resistance did not change (Figure 2I). A sintering temperature of 240 °C was maintained for 1 h to enable low‐resistance Ag electrodes. Increasing the sintering temperature, up to 300 °C, has been shown to lower AgNP resistivity.^22^ However, the PMMA layer required for transfer to elastomer may be damaged and prevent transfer due to excessive exposure to high temperatures. The resulting AgNP electrode thickness was 4.1 µm with a width of 189 µm (Table 2). The overall resistance of the thin (6.8 µm) AgNP electrode on PI was less than 25 Ω. This is lower than that of the previously reported AJP‐enabled freestanding interconnects despite those being 15 µm thick.^21^ The resistance remained higher due to the use of a composite layer of PI and AgNP as the conductive paths. Here, the optimized fabrication process uses separate layers of PI and highly conductive AgNPs to simultaneously create mechanically stable and low‐resistance electronics.

The dielectric layer is printed with the same PI ink on top of the patterned PI/AgNP structure. The resulting profile with a dielectric layer is shown in Figure 2J. Although a thin dielectric layer provides high sensitivity and high capacitance for a given area, thin PI inks do not fully cover the AgNP profiles, despite multiple passes, and the two AgNP electrodes become electrically connected. Fewer passes with thick PI inks perform better than more passes with a thin PI ink. Although a similar thickness and sufficient insulation with PI may be achieved by increasing passes for both PI inks, a thin PI ink causes loss of feature, as indicated in Figure 2B,C. Also, thin PI inks fail to cover the rough AgNP surface with fewer passes due to this spreading. Here, 4 passes of the 4:1 PI ink prevent electrode shorting, while providing ≈2.5 µm thick layer. However, this thickness varies due to the curved profile of AgNP traces. This layer is cured and plasma‐treated, and printing the top AgNP electrode with a stage temperature of 80 °C completes sensor fabrication. The final sensor has a thickness of 12.4 µm and a width of 306 µm, as shown in Figure 2K (Table 2). Sensor capacitances range between 60 and 80 pF, which correlates well with measured dielectric thickness and AgNP electrode area.

### 2.3 Sensor Design and Mechanics

**Figure 3:**
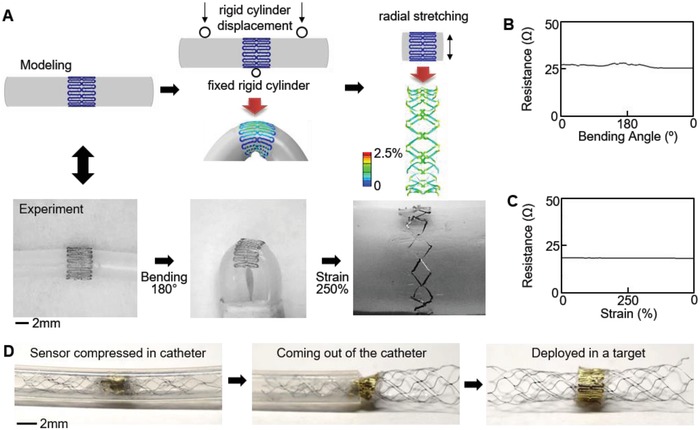
Mechanics modeling, fabrication, and reliability of a printed biosensor for a catheter‐based deployment in a target vessel. A) Design of a highly stretchable sensor via finite element modeling and comparison with the fabricated sensor. B,C) Measured electrical resistance of the printed sensor under a cyclic bending and radial stretching, showing negligible change of resistance under both loading conditions. D) Catheter‐based deployment of the fabricated sensor in a target blood vessel, enabled by the highly flexible and stretchable structure.

These optimized printing parameters yield a highly stretchable and flexible design. Based on printed trace widths, optimization of spacing between adjacent traces to prevent overlapping results in Ag filling fraction of 39% in a 3 × 10 mm^2^ rectangle (Figure S6, Supporting Information). Mechanical properties of these printed films are also highly important for sensor functionality. The measured moduli of printed PI and AgNP film are 1.5 and 52 GPa, respectively. The lower modulus of the printed PI improves sensitivity due to easy deflection upon flow introduction. A serpentine design achieves the high flexibility and stretchability for implantation in contoured neurovessels. Finite element analysis indicates a stretchability of 250% and 180° bending about a 1 mm diameter prior to resistance increase occurring near 2.5% strain in Ag (**Figure** **3**A).^26^ The mechanics are experimentally validated with a radial expansion test and bending test (details in Note S1 and Figure S7, Supporting Information). Quantitative mechanical tests (Figure 3B,C) show negligible change of electrical resistance when subjected to 250% strain and 180° bending followed by release. These conditions meet requirements to deploy the sensor with the stent via the standard catheter procedure. Figure 3D shows the sequential process of deployment of the integrated sensor system by a catheter. The extremely small form factor and mechanical compliance of the sensor allow seamless integration with an expanded stent followed by compression and deployment without damage.

### 2.4 Wireless Readout Method Design and Optimization

**Figure 4:**
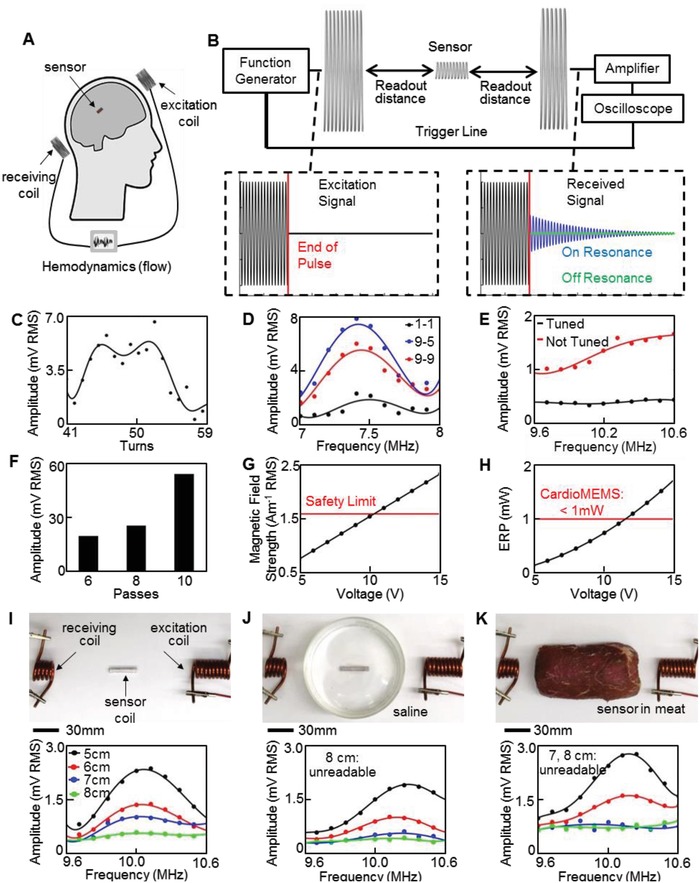
Parametric study and optimization of an inductive coupling method. A) Schematic overview of the batteryless wireless system with an implantable flow sensor and two external antenna coils. B) Equipment overview and example excitation signals. Received transient sensor signals differ for on and off resonance cases. C) Experimental validation of optimal number of sensor coil turns to increase amplitude at resonance. D) Signal amplitude according to the frequency showing the largest resonance with a 9‐turn excitation coil and 5‐turn receiving coil (blue curve). E) Minimized noise due to tuning of antenna coils at the frequency range of interest. F) Signal amplitude showing that sensors with thicker Ag (multiple passes) transmitting a larger resonant peak. G) Comparison of excitation magnetic field strength 1 cm away from excitation coil and averaged over a projected area of 20 × 20 cm^2^ with safety limit of 1.6 A m^−1^. H) CardioMEMS HF system operates with an ERP greater than that used in this work with 5 V. I) In vitro test of the sensor coil and ceramic capacitor in different conditions, including frequency sweeps of sensor coil through air, readable up to 8 cm, J) sensor coil readable up to 7 cm when placed in saline solution, and K) sensor coil with the detection range to 6 cm in meat.

For an implantable system with wireless monitoring of hemodynamics, we utilize and optimize an inductive coupling method that offers circuit‐free design. **Figure** **4**A captures the overview of the proposed system for wireless quantification of hemodynamics in cerebral aneurysms. The wireless readout method applies inductive coupling principles between a sensor coil in a flow‐diverter system and two external coils to record transient signals (Figure 4B). A capacitive sensor, combined with an inductive coil, forms a circuit with a resonant frequency defined as

(1) $$f=1/\left(2\pi \sqrt{LC}\right)$$

where *L* is inductance and *C* is sensor capacitance. This readout method, compared to the frequency domain method of observing impedance changes, has been proven to achieve longer readout distances.^16^ The root‐mean‐square (RMS) amplitudes of transient signals after excitation are compared to identify sensor resonance. During the measurement, the overall readout range is defined as the distance between the end of the sensor coil and the nearest external coil. A function generator, low‐noise amplifier, and oscilloscope form the system electronics (Figure S8, Supporting Information). Analytical study plays a key role in understanding the key parameters of resonant frequency, quality factor, and power transfer efficiency to optimize a sensor coil design. We investigated the number of turns, length, and wire diameter for the sensor coil, while the coil diameter was fixed to 3.5 mm for the strictest cerebral aneurysm applications; the coil pitch was determined by turns and length.^14^ Increasing conductive cross‐sectional area for a given material improves quality factor and transfer efficiency due to a decrease in resistance. This improvement is only limited by the skin effect phenomenon. Analytical and experimental studies use a 100 µm diameter wire. The optimization results for sensor quality factor, coupling coefficient, and transfer coefficient indicate that a critical value for number of turns exist to achieve a balance between quality factor and power transfer efficiency (Figure S9A–C, Supporting Information). Experimental tests validate this conclusion through a comparison of RMS values at resonance while varying the number of turns (Figure S9D,E, Supporting Information). As a result, coils with 44 to 53 turns and 30 mm in length show higher RMS values, which enables a longer readout distance (Figure 4C). Resulting coils have analytical inductances between 1.5 and 1.9 µH and induced power is measured as 1.11 and 1.34 mW for 44 and 52 turns, respectively. These tests use 150 to 220 pF capacitances to achieve low resonant frequencies between 10.2 and 12.2 MHz. Changing sensor capacitance does not impact induced power, but drops the quality factor, yielding a less pronounced resonance peak (Figure S9F, Supporting Information).

A similar procedure investigates external coil parameters to enhance readout distance. Coupling, transfer efficiency, and induced current are studied while varying coil diameter and number of turns. Coupling and transfer coefficients indicate an optimal coil diameter, whose value depends on the distance between coils (Figure S10A,B, Supporting Information). Computational results indicate that a higher number of turns in the excitation coil increase the induced current, and experimental tests verify the result (Figures S10C and S11, Supporting Information). Experimental frequency sweeps validate the analytical results by varying number of turns from 1 to 20 and coil diameter between 20 and 60 mm. Single turn coils achieve longer readout distances for increasing diameters. However, multiturn coils perform better with a 20 mm diameter in terms of noise and readout distance. Although received noise increases due to additional turns, as indicated by a prior study, RMS values at resonance are larger for specific turn combinations.^16^ As a result, an excitation coil with 9 turns and a receiving coil with 5 turns provide the maximum difference between resonant peak and noise (Figure 4D). Here, we also investigate tuning the external coils to resonate near sensor resonance. Tuning both coils approximately doubles power transfer at resonance between the excitation and sensor coil from 1.34 to 2.56 mW at a 3 cm distance. A more consistent noise level in the received signal also occurs via tuning (Figure 4E). Printing parameters of capacitive sensors also significantly affect readout distance due to equivalent series resistance (ESR) of the capacitor. Electrical resistance of the individual AgNP electrodes contributes to ESR and attenuates the sensor's transient signals. Increasing the thickness of the electrodes with multiple passes reduces resistance and significantly improves signal amplitude (Figure 4F). In the present work, the number of AgNP passes was limited to 10, achieving a thickness of 4 µm when printed on a PI layer. Future work may investigate printing thicker and flatter Ag electrodes to reduce resistance and increase readout distances. To verify compliance with safety limits of sensor implantation, a computational model estimates RMS magnetic field strength for comparison. Our system shows an average strength of 0.78 Am^−1^ at 10.2 MHz, which is well below the safety level of 1.6 Am^−1^ averaged over 30 min (Figure 4G).^27^ The model also indicates the RMS electric field strength to be 6.83 V m^−1^, significantly lower than the limit (80.8 V m^−1^). System settings here result in lower averages over a 30 min period due to short excitation periods. The proposed system uses about 5 times smaller effective radiated power (ERP = 0.19 mW) than that from the commercial device (CardioMEMS; ERP = 1 mW) with the longest readout distance of 20 cm (Figure 4H).^28^, ^29^ These results suggest that the magnetic field of our wireless system can be further amplified to increase the interrogation range, though closer examination in the future may be required regarding specific absorption rates and localized exposure. Experimental tests through air, saline, and meat (beef) validated the system parameters to indicate readout distance limits of the sensor coil connected to a ceramic capacitor. Figure 4I shows an experimental setup that includes a sensor coil in air, which results in the maximum working distance up to 8 cm. Figure 4J simulates a sensor coil, embedded in a blood vessel, which includes a saline solution (volume: 225 mL). The maximum readout distance with saline is 7 cm due to signal attenuation. Finally, implantation of a sensor coil in a human tissue is simulated by completely encapsulating the sensor coil in meat (cross‐section area: 6 × 6 cm^2^; Figure 4K). When embedded in the raw beef, the amplitude of the transient response and noise increases, resulting in a maximum communication distance of 6 cm. These limits indicate feasibility of the system for a coil diameter as small as 3.5 mm. It should be noted that human brain tissue produces a magnetic field^30^ (<0.01 µT) and our experiments were conducted in an unshielded environment where ambient noise was near 0.1 µT.^31^ On the other hand, the excitation coil of the wireless system produced ≈1 µT, which can neglect the effects of magnetic fields from the brain tissue and ambient noise.

### 2.5 In Vitro Demonstration of Device Functionality

**Figure 5:**
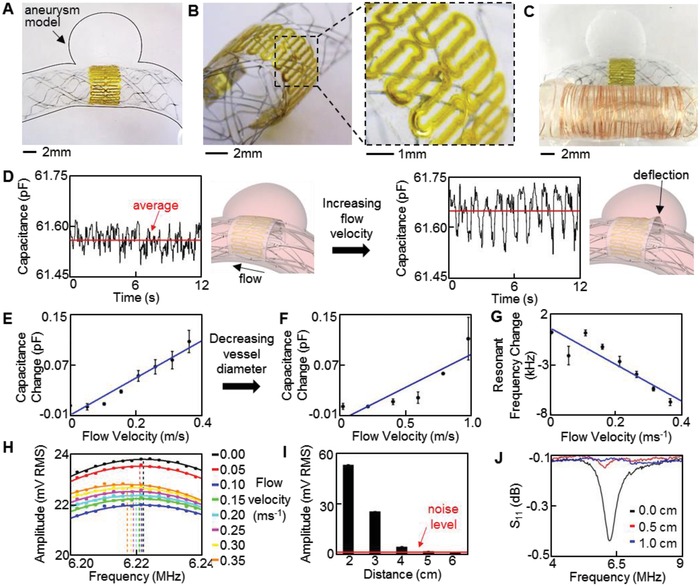
In vitro study of the printed sensor performance in a biomimetic blood vessel model. A–C) Photos of a medical stent‐integrated sensor in an aneurysm model, showing A) a zoomed‐out overview of the sensor, B) tilted, zoomed‐in view of the ultrathin, low‐profile sensor, and C) a copper coil connected sensor for wireless monitoring of hemodynamics in the biomimetic model. D) Pulsatile flow velocity captured by the sensor; average sensor capacitance increases with flow velocities from 0.05 (left) to 0.35 m s^−1^ (right). Illustration (right) indicates mechanism of capacitance change due to increased flow into aneurysm. E,F) Average capacitance change with the increase of E) flow velocity in the 5 mm vessel and F) higher flow velocities in the 3.5 mm vessel. G) Decreased resonant frequency with increased flow velocity. H) Continuous frequency sweeps, showing the shift in resonant frequency. I) Decreased peak amplitude at resonance with the increased distance; 6 cm interrogation distance is achieved before a resonance peak is not observable. J) Lowered readout distance (less than 1 cm) in monitoring of resonant frequency with a VNA, indicating superiority of the optimized wireless inductive coupling method.

Comprehensive study and optimization of the fully passive, inductive coupling method capture the feasibility of our system for wireless monitoring of flow in aneurysms (**Figure** **5**). A set of in vitro experiments demonstrate the sensor performance in monitoring of both capacitance change and associated resonance shift. A pulsatile blood pump (Harvard Apparatus) simulates blood flow through a polydimethylsiloxane (PDMS) aneurysm model with 5 mm diameter parent blood vessel and sac (Figure S12, Supporting Information). The sensor and stent devices are aligned in front of the sac opening (Figure 5A), and allow flow to enter and exit the aneurysm. Leaving the sensor in a nonstretched form increases deflection and improves sensitivity, as deflection is the primary source of capacitance change by decreasing the dielectric thickness. The nonstretched form also increases the percentage of the capacitive pattern in the region of deflection. The AgNP electrodes are twice thicker and have a 27 times higher modulus than the PI layer (Table 1). During the flow‐induced bending, the AgNP electrodes act as rigid plates, while compressing the softer PI layer near the bending regions. This mechanism was validated by a finite element analysis of deflection of the sensor pattern, which indicated the highest strain in the dielectric PI layer (Figure S13, Supporting Information). The amplitude of deflection corresponds to the average dielectric thickness change. Overall, this result well agrees with the previous work that related sensor deflection to capacitance change with a computational fluid model.^15^ The ultrathin, low‐profile form factor of the sensor (Figure 5B) avoids disruption to normal hemodynamics. For wireless monitoring, a copper coil is externally connected to the capacitive sensor (Figure 5C). Mean flow velocity in the 5 mm diameter blood vessel is varied from 0 to 0.35 ms^−1^ at 0.05 ms^−1^ intervals. Initial sensor capacitance is 61.53 pF and increases to 61.63 pF at the maximum flow velocity (Figure 5D). This increase in capacitance is a result of flow entering the aneurysm sac and deflecting the sensor (Movie S3, Supporting Information). Figure 5D illustrates this mechanism of capacitance change. The sensor captures the pulsatile nature of the blood flow, and average capacitance increases when fluid flows through the vessel (Figure S14, Supporting Information). Figure 5E shows an average capacitance increase, indicating an overall sensitivity of 0.29 pF m^−1^ s. Typical intracranial blood flow velocities range between 0.5 and 1.0 m s^−1^, thus the sensor was tested in a highly contoured 3.5 mm diameter vessel to increase flow velocity (Figure S15, Supporting Information).^32^ Sensor capacitance increased with increasing mean flow velocity from 0 to 1.0 m s^−1^, but showed a lower sensitivity of 0.084 pF m^−1^ s (Figure 5F). The lowered sensitivity to parent vessel flow velocity can be explained by considering flow into the aneurysm sac, which is the flow monitored by the sensor (Figure S15C, Supporting Information). It is important to distinguish the difference between the parent vessel flow and the aneurysm neck flow. In this study, parent vessel flow velocity is varied as we have previously shown that increasing parent vessel flow velocity also increases aneurysm neck flow velocity.^15^ The 3.5 mm diameter parent vessel contained an aneurysm with a neck diameter of 2 mm, while the 5 mm diameter parent vessel contained an aneurysm with a neck diameter of 4 mm. This decrease in neck diameter corresponds with a decrease in aneurysm neck flow for a given parent vessel flow velocity.^33^, ^34^ Thus, comparing the sensor performance in the two aneurysm geometries confirms that the low‐profile sensor is sensitive to aneurysm neck flow, the criteria for flow diverter treatment monitoring, and avoids undesired noise resulting from the parent vessel flow. The increase in average capacitance lowers the resonant frequency to enable wireless detection. Although the sensor will deform with the stent during implantation, an initial resonant frequency can be determined to calibrate the system for wireless detection. Frequency sweeps are continuously performed to monitor flow velocity and a 7.5 mm diameter inductive coil completes the sensor electronics.

In this work, a larger diameter (7.5 mm) coil was used to improve validation of the capacitive sensor and wireless telemetry due to increased resistance at wired connections between the capacitive sensor and coil. However, future work will involve development of a smaller implantable coil directly integrated with the capacitive sensor to achieve a complete sensor package. Calculations indicate an analytical inductance of 9.1 µH and resonance of 6.6 MHz but experimental tests show ≈6.2 MHz. The discrepancy may result from manual wrapping of the copper coil and from the wire connections between the sensor and coil. Details of transient signals were recorded for a printed sensor and additional frequency sweeps while monitoring flow appear in Figures S16 and S17 (Supporting Information), respectively. Figure 5G determines the sensitivity of the wireless flow sensor (−18.9 kHz m^−1^ s) based on the resonant frequency according to various flow rates. Figure 5H summarizes a set of frequency sweeps used to determine resonant frequency, indicating an overall decrease in resonant frequency as flow velocity increases. Wireless monitoring indicates a 6.5 kHz shift for a 0.1 pF change, correlating well with the analytical shift of 5.9 kHz. The maximum readout distance of printed sensors is up to 5.5 cm in air (Figure 5I), which can be further extended via increasing of the number of Ag passes. For comparison to the optimized time domain measurement, a frequency domain measurement is performed while distance between a reader coil and sensor coil is increased. Here, a vector network analyzer (VNA; Tektronix TTR506A) records the scattering parameter S_11_ through single‐turn, 2 cm diameter reader coil (Figure 5J). The maximum distance achieved by the frequency domain measurement is 0.5 cm, significantly lower than that needed for practical applications. Moreover, we operate the transient inductive coupling system at approximately half the transmitting power of the safety limit, allowing for immediate improvement and further reduction of the coil size.

**Table** **3** compares the key parameters and resultant readout distance of reported wireless sensors that use inductive coupling methods.^6^, ^16^, ^35^, ^36^, ^37^, ^38^ Our system and optimized coils offer significant improvement in both implantation size and readout distance compared to other systems. Overall, the newly designed device via AJP and optimization of wireless telemetry demonstrate the most applicable performance among the reported inductive coupling methods, in terms of the ratio of wireless readout distance and sensor cross‐section area. It should be noted that the planar, stacked coils (Fonseca, 2016 and Abbott, 2017) used much higher magnetic fields than our system.^37^, ^38^ The enhanced wireless distance in this work allows for integration with a cerebral aneurysm sensor for monitoring hemodynamics in the brain, which requires an average readout distance of 3–6 cm.^17^ The current limitation of this study is the integration of an implantable coil with the stent and sensor system to achieve a complete implantable package. Currently, we are developing methods for integration of an implantable, inductive coil with the existing stent and flow sensor. The effect of the properties of blood on the sensor and implantable coil, such as capacitive shorting between coil turns, will be further investigated. Following completion of the sensor system, in vivo experiments will be performed. Future work will also involve hemocompatibility and biocompatibility tests of the sensor system, as the toxicity of AgNPs will be addressed.^39^ However, in this work, we confirmed that the biocompatible PI layer^40^ fully encapsulated the completely sintered AgNP films, which was verified by a Raman analysis (Figure S18, Supporting Information). Thus, no direct interaction between Ag films and human tissues is expected in this system.

**Table 3:** Parameter comparison of wireless sensing devices using inductive coupling

Reference	Ratio of readout distance‐areaa)	Type	Readout distance [cm]	Sizeb)	R. freq.c) [MHz]	Quality factor	Induc‐tance [µH]	Area [mm^2^]
This work	2.4	Solenoid coil	5.5 (air)	15 mm (*l*), 7.5 mm (*d*), 0.1 mm (*t*)	6.2	153	9.1	7.85 × 10^−3^
(Park et al. 2016)	0.4	Planar coil in stent	0.2 (air)	3 × 3 mm^2^, 0.15 mm (*t*)	200	10	0.839	2.25 × 10^−4^
(Chen et al. 2014)	3.8	Planar, stacked coils	1.5 (air)	4 × 4 mm^2^, 0.1 mm (*t*)	1000d)	–	–	–
(Chen et al. 2010)	1.3	Planar coil	2 (air)	4 mm (*d*)	350	30	0.057	–
(Brox et al. 2016)	1.6	Gold‐coated stent	2.75 (air)	20 mm (*l*), 5 mm (*d*)	50.7	24	0.53	4.42 × 10^−3^
(Fonseca et al. 2006)(Abbott 2017)	2.9	Planar, stacked coils	20e) (air/implant)	15 mm (*l*), 3.4 mm (*w*), 2 mm (*t*)	30–50	65–77	–	2.88 × 10^−3^

a) It defines the ratio of wireless readout distance and cross‐sectional area of the sensor. Our work shows the best performance among the reported inductive coupling methods

b) Note that some references have limited information about the sensor size. Unit expression: length (*l*), diameter (*d*), width (*w*), and thickness (*t*)

c) R. freq.: resonance frequency

d) High frequency signals significantly attenuate in tissue and limit readout distance when implanted

e) Achieved with double the system power used here.

## 3 Conclusion

Collectively, this work provides a comprehensive understanding of AJP techniques for a highly stretchable, multilayer electronic system on soft substrates. Unlike conventional microfabrication with top‐down approaches, the process reported here is a fully additive, easily scalable method while enabling seamless integration with soft materials. Parametric study of aerosol materials summarizes the critical factors in thin, multilayer electronics printing. Mechanical studies display the highly stretchable and flexible design of the sensor. Deploying the integrated sensor and stent system indicates compatibility with existing procedures. Optimizing the wireless detection scheme shows feasibility of reading this passive, implantable sensor for continuous, wireless cerebral aneurysm monitoring and provides an alternative to repetitive Doppler measurements and invasive angiographic procedures. Experimental studies confirm performance of the low‐profile flow sensor and wireless interrogation. The novel fabrication method and advanced material study, described here, are widely applicable for rapid development of wireless and stretchable electronics.

## 4 Experimental Section

*Fabrication of Sensor*: Sensor fabrication uses an aerosol jet printer for all sensor layers. A sacrificial layer of PMMA was spin‐coated on a glass slide at 4000 RPM for 30 s and baked at 180 °C for 2 min. PI ink was prepared by mixing PI‐2545 precursor with NMP in a 4:1 ratio. A patterned layer of PI was deposited via aerosol jet printer in 3 passes using a 300 µm nozzle diameter. The printed PI was cured at 240 °C for 1 h. Next, 10 passes of silver were printed onto the PI using AgNP ink and a 200 µm nozzle diameter. The AgNP layer was then sintered at 240 °C for 1 h. The dielectric layer of 4 passes of PI was printed and cured using the same parameters as before. The final silver layer was printed and sintered with identical parameters. The PMMA layer was dissolved in an acetone bath prior to transfer to a thin layer of Ecoflex gel spin coated at 1500 RPM on a PVA film. The printing process is illustrated in Figure S1 (Supporting Information). Copper wire was attached to sensor contact pads with silver paint prior to encapsulation with Ecoflex. The top and bottom of the sensor were attached to a stent with a small amount of Ecoflex.

*Finite Element Modeling*: Finite element analysis results for geometry and boundary conditions are shown in Figure 2D. All simulations were performed in Abaqus (Dassault Systems). The radial stretching analysis used a deformable, hollow cylinder to expand the sensor pattern. Sensor layers included were elastomer encapsulation, two PI layers, and two Ag layers. The cylinder was radially displaced to achieve 250% strain. The bending model employed a solid, deformable cylinder that was deformed by three rigid cylinders to achieve 180° bending. The bending radius was 0.5 mm. A tie constraint between the support cylinder and inner sensor encapsulation ensured uniform contact.

*Experimental Measurement of Resonant Frequency*: The wireless readout method applied inductive coupling principles between a sensor coil in a flow‐diverter system and two external coils to record transient signals and measure sensor resonance. An excitation external coil (8 AWG copper wire), connected to a function generator (Keysight), transmitted a pulse of 20 sine cycles at a specified frequency and maximum amplitude (10 peak‐to‐peak voltage). The readout coil, attached to a low‐noise amplifier (Model ZFL‐1000LN+, Mini‐Circuits) and oscilloscope (Tektronix), recorded the signals from the excitation coil and sensor circuit. The oscilloscope was triggered to record the transient signal by syncing it with the function generator. The function generator and oscilloscope were controlled by a custom Matlab program to record frequency sweep results. A maximum transient response from the sensor coil occurred when the excitation frequency matched the sensor's resonance frequency. The frequency sweep was used between 0.001 and 0.1 MHz steps to identify resonance. Transient signals were processed by the program to remove signal drift, identifying an appropriate RMS window that is 6 cycles in length, and calculating an RMS value for that frequency. The RMS values were saved and a quadratic curve was fit to the frequency sweep. The maximum amplitude on the fitted curve was located and the corresponding frequency was identified as its resonant frequency. During the measurement, three coils were axially aligned and the overall readout range was defined as the distance between the end of the sensor coil and the nearest external coil.

*Analytical Study of Wireless System*: An analytical study optimized the system parameters, including coil length (*l*), coil diameter (*d*), and number of turns (*N*), which was used to improve the wireless readout distance via inductive coupling. Both internal (*i*) and external (*e*) coils were studied. Sensor inductance (*L*) was calculated with correction coefficients (Equation (2)), which offered more accurate estimate.^41^ The coefficient *k*_m_ is a mutual inductance correction for a round wire and *k*_s_ is a self‐inductance correction.^42^, ^43^ Inductance along with sensor capacitance (*C*) defined sensor resonance (*f*). Low resonance frequency was preferred to allow a higher magnetic field, while reducing tissue absorption from a sensor during wireless telecommunication, which enables a long readout distance.^27^ Specific absorption rate was a measure of safety when radio frequency was applied and known to scale with frequency by *f* ^2.15^.^44^ The readout distance of the inductive coupling method depends on the sensor's quality factor (*Q*), function of inductance, capacitance, and resistance (*R*) (Equation (3)). The quality factor determined the speed of response decay, efficiency of power transfer at resonance, and bandwidth. A larger quality factor resulted in a more detectable transient response. Mutual inductance (*M*) (Equation (4)) and coupling coefficient (*k*) (Equation (5)) also played key roles in power transfer and they depended on the separation distance (*z*).^45^ More efficient power transfer between the sensor and receiver coils improved the readout distance. Finally, to quantify the impacts of quality and coupling on power transfer, overall transfer coefficient, (*Π*) (Equation (6)) and transfer efficiency (η) (Equation (7)) were defined.^46^ The maximum readout distance was determined by varying those parameters

(2) $$L=\frac{\mu N^{2}\pi d^{2}}{4l}-\mu \frac{d}{2}N(k_{s}\;+k_{m}),\;\mathrm{where}\;\mu =\mathrm{permeability}$$

(3) $$Q=\frac{1}{R}\sqrt{\frac{L}{C}}$$

(4) $$M=\frac{\mu \pi N_{i}N_{e}d_{i}^{2}d_{e}^{2}}{16\sqrt{d_{i}^{2}+z^{2}}}$$

(5) $$k=\frac{M}{\sqrt{L_{i}L_{e}}}$$

(6) $$\Pi =\frac{k\sqrt{Q_{i}Q_{e}}}{1+k^{2}Q_{i}Q_{e}}$$

(7) $$\eta =\frac{k^{2}Q_{i}Q_{e}}{1+k^{2}Q_{i}Q_{e}}$$

3D finite element analysis (COMSOL) calculated the induced current between the sensor coil and excitation external in air. The computational model used 100 µm diameter copper wire and 8 AWG copper wire for the sensor and excitation coil, respectively. Induced current in the sensor coil was calculated while varying antenna turns. For comparison with safety limits, a second finite element model of the excitation coil simulated magnetic field strength while varying input voltage for comparison with safety limits. Additionally, to compare with a commercial implantable sensor, ERP was calculated using electric field strength (*E*) and distance from the source (*z*) using Equation (8), 28

(8) $$\mathrm{ERP}=\frac{{\left(Ez\right)}^{2}}{30\times 1.64}$$

*In Vitro Experiment*: A PDMS mold of an aneurysm and blood vessel was created to simulate flow. The sensor was placed over the aneurysm neck and the mold was sealed with additional PDMS. A pulsatile pump generated flow through the model at 60 strokes per minute. Flow volume was adjusted to achieve the desired flow rate. For wired measurements of capacitance, an LCR meter that measures inductance (L), capacitance (C), and resistance (R) (B&K Precision) was connected to the sensor. For wireless monitoring, a copper coil was connected to the capacitive sensor. Two external coils were aligned with the sensor coil and resonant frequency was monitored.

## Conflict of Interest

R.H. and W.‐H.Y. are the inventors on a pending US patent application.

## Supporting information

Supplementary

Supplementary

Supplementary

Supplementary
