# Supplementary material for: Fully Printed, Wireless, Stretchable Implantable Biosystem toward Batteryless, Real‐Time Monitoring of Cerebral Aneurysm Hemodynamics
Source: Adv Sci (Weinh). 2019 Aug 7;6(18):1901034. doi: 10.1002/advs.201901034 (PMC6755526; doi:10.1002/advs.201901034)
Supplement: Supplementary file 1 — Supplementary [file ADVS-6-1901034-s002.pdf]

Copyright WILEY-VCH Verlag GmbH & Co. KGaA, 69469 Weinheim, Germany, 2019.

ADVANCED SCIENCE
Open Access

Supporting Information

for *Adv. Sci.,* DOI: 10.1002/advs.201901034

Fully Printed, Wireless, Stretchable Implantable Biosystem toward Batteryless, Real-Time Monitoring of Cerebral Aneurysm Hemodynamics

*Robert Herbert, Saswat Mishra, Hyo-Ryoung Lim, Hyoungsuk Yoo, and Woon-Hong Yeo**

Supporting Information

Fully printed, wireless, stretchable implantable biosystem towards batteryless, real-time monitoring of cerebral aneurysm hemodynamics

*Robert Herbert, Saswat Mishra, Hyo-Ryoung Lim, Hyoungsuk Yoo, and Woon-Hong Yeo**

Mr. R. Herbert, Dr. S. Mishra, Dr. H. Lim, Prof. W.-H. Yeo
George W. Woodruff School of Mechanical Engineering, Institute for Electronics and Nanotechnology, Georgia Institute of Technology, Atlanta, GA 30332, USA

Prof. H. Yoo
Department of Biomedical Engineering, Hanyang University, Seoul 04763, South Korea

Prof. W.-H. Yeo
Wallace H. Coulter Department of Biomedical Engineering, Parker H. Petit Institute for Bioengineering and Biosciences, Neural Engineering Center, Center for Flexible and Wearable Electronics Advanced Research, Institute for Materials, Georgia Institute of Technology, Atlanta, GA 30332, USA

E-mail: whyeo@gatech.edu (W.-H. Yeo)

Keywords: aerosol nanoparticles, aerosol jet 3D printing, stretchable hybrid electronics, batteryless wireless monitoring, hemodynamics

Supporting Note S1. Mechanical Stretching and Bending Test

A hollow elastomer tube is attached to a syringe pump and valve via a micro pipette adapter. The elastomer tube is created by spin coating Ecoflex and wrapping it around a tube, using additional Ecoflex to seal it. The sensor is placed around the tube and the syringe is used to inflate the hollow tube, using a clamp to seal the other end of the tube. Copper wire is attached with silver paint to monitor resistance via a multimeter (Keithley). The bending test consists of a solid elastomer tube. The sensor is wrapped around the tube and connected to copper wires. Two glass slides are connected to the tube with medical tape and spaced 1 mm apart. The slides are joined with polyimide tape. The slides are rotated towards each other to provide 180º bending to the sensor and a multimeter records resistance.

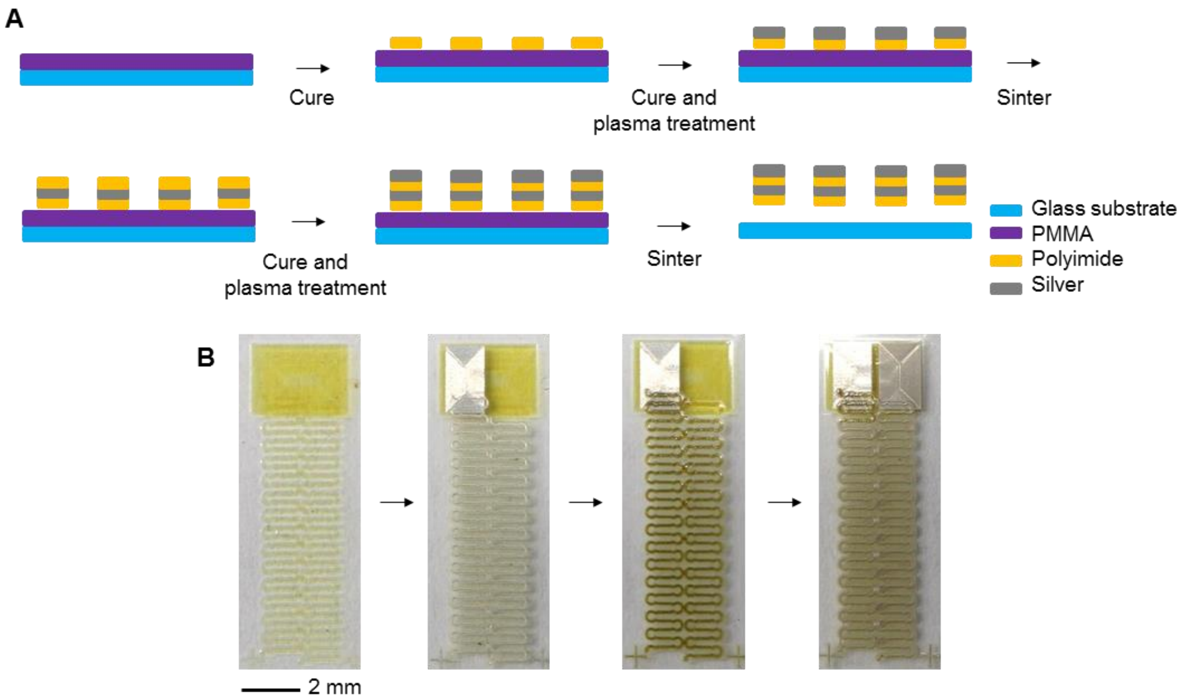

Figure S1. Illustration of sensor fabrication via AJP. A) A sacrificial PMMA layer is spin coated on a glass substrate. A PI layer is printed for transferring and then cured. AgNP ink is then printed and sintered on a plasma treated PI layer, followed by printing of a second PI and Ag layer. PMMA is then dissolved in an acetone bath for transfer to elastomer. B) Photos of material deposition from base PI layer to top AgNP layer.

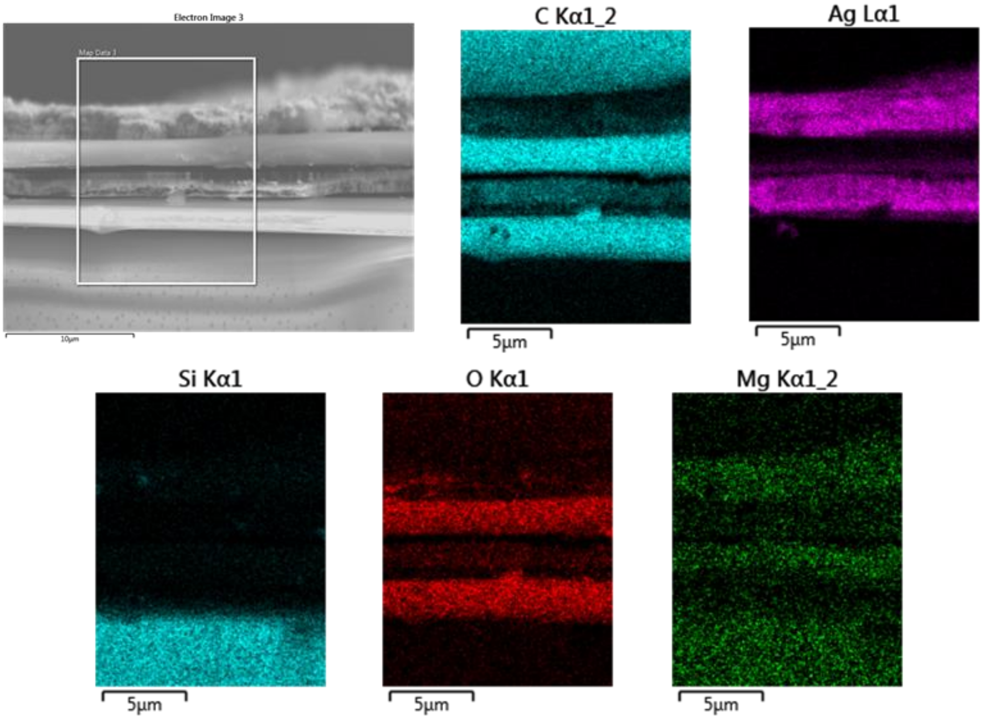

Figure S2. Scanning electron microscopy with energy dispersive spectroscopy characterizes the composition of the sensor layers and indicates AgNP layers with dielectric layer in-between.

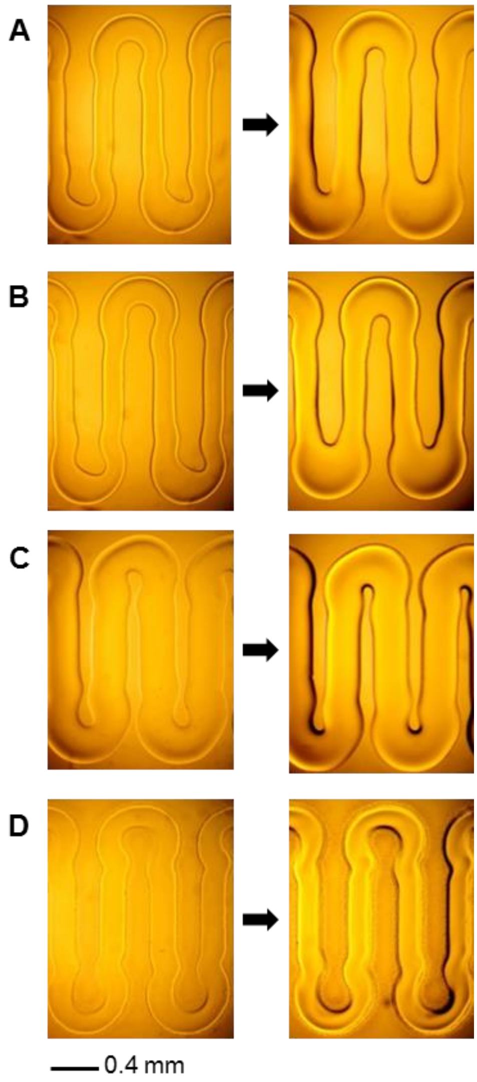

Figure S3. Printed PI from 1 pass to 5 passes at PI:NMP ratios of A) 1:1, B) 2:1, C) 3:1, and D) 4:1. The higher ratio inks maintained pattern shape and showed limited spreading.

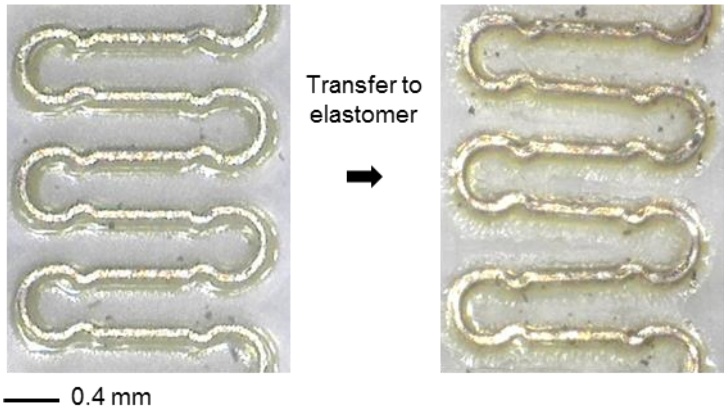

Figure S4. Sensor pattern before and after transfer to elastomer.

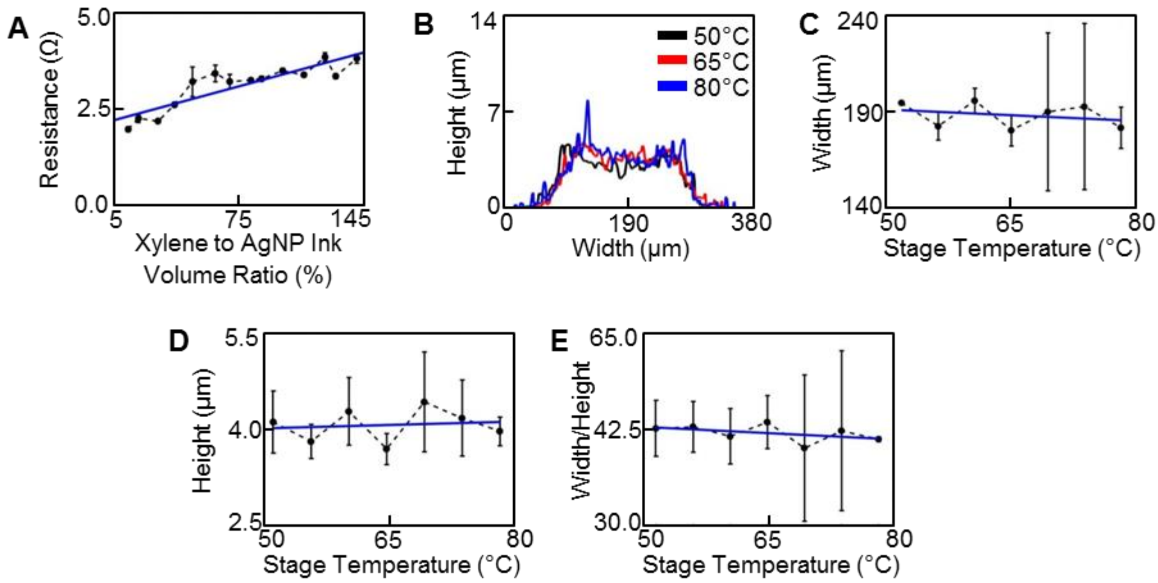

Figure S5. Characterization of AgNP traces. A) Adding xylene to AgNP ink minimally increases resistance of printed patterns. B) Profiles of AgNP film at different stage temperatures. Higher temperature leads to large spikes on the surface. C) Higher stage temperature provides a small decrease in width. D) Thickness shows minor increase with stage temperature. E) Width to height ratio shows minor decrease as stage temperature increases.

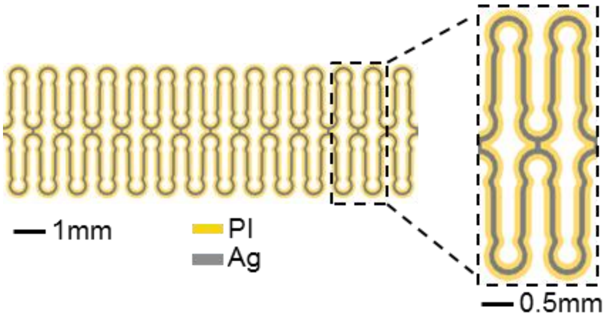

Figure S6. Sensor pattern showing Ag deposited on PI to achieve a unit cell area coverage of 39%.

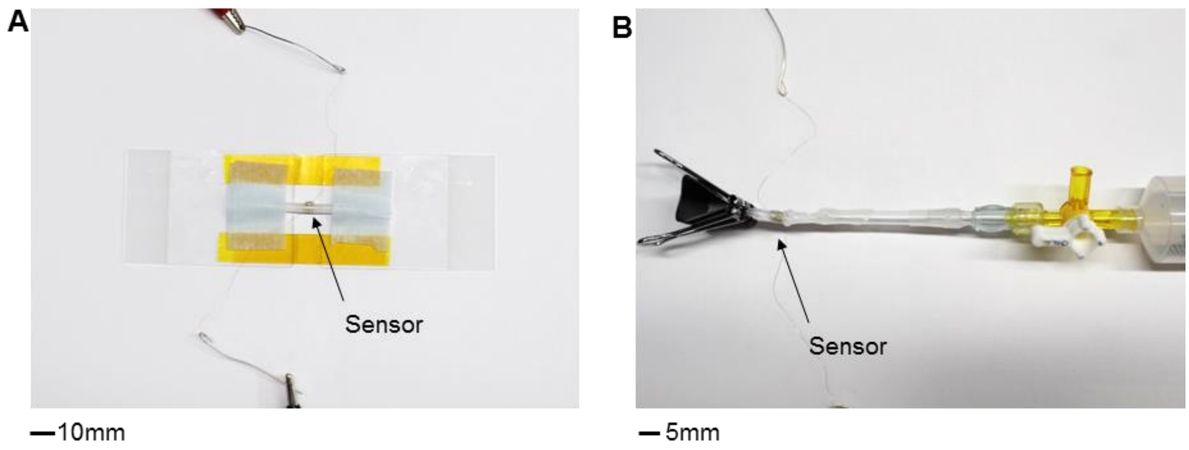

Figure S7. Experimental testing of mechanics. A) Experimental setup for bending. The sensor is attached to the elastomer tube and rotation of the glass slides results in bending. B) Experimental setup for radial stretching of sensor. The syringe inflates the hollow elastomer tube for expansion.

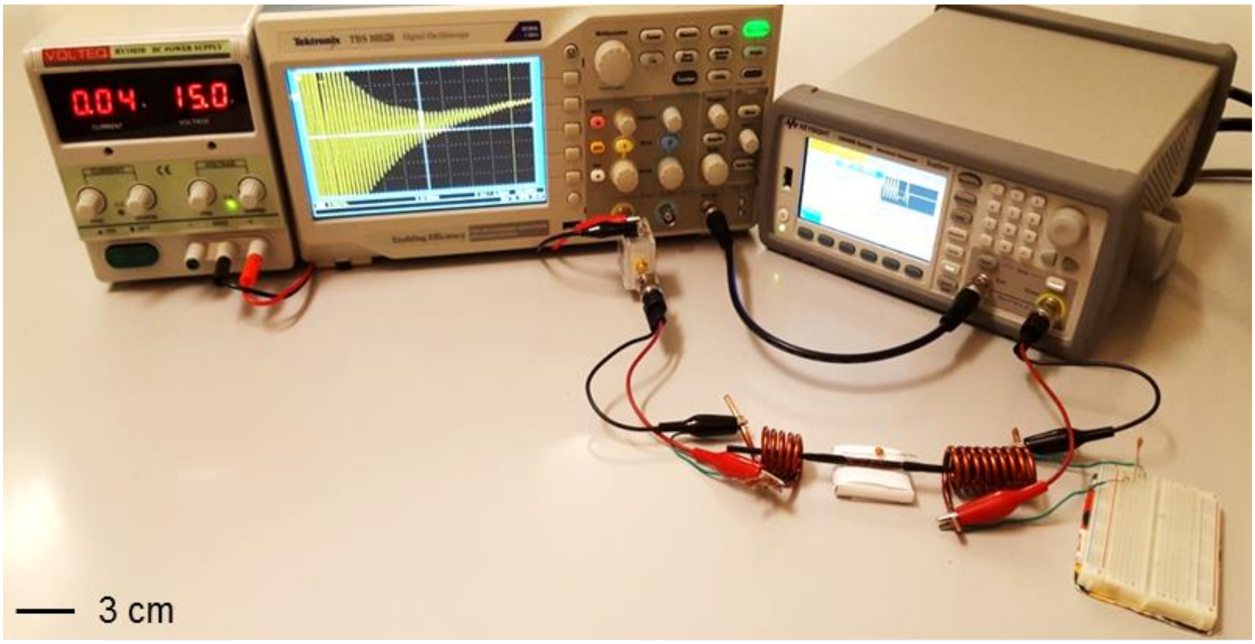

Figure S8. Wireless experiment setup using DC power supply, oscilloscope, and function generator. Test measurement through air. The transient response of the sensor is displayed on the oscilloscope screen.

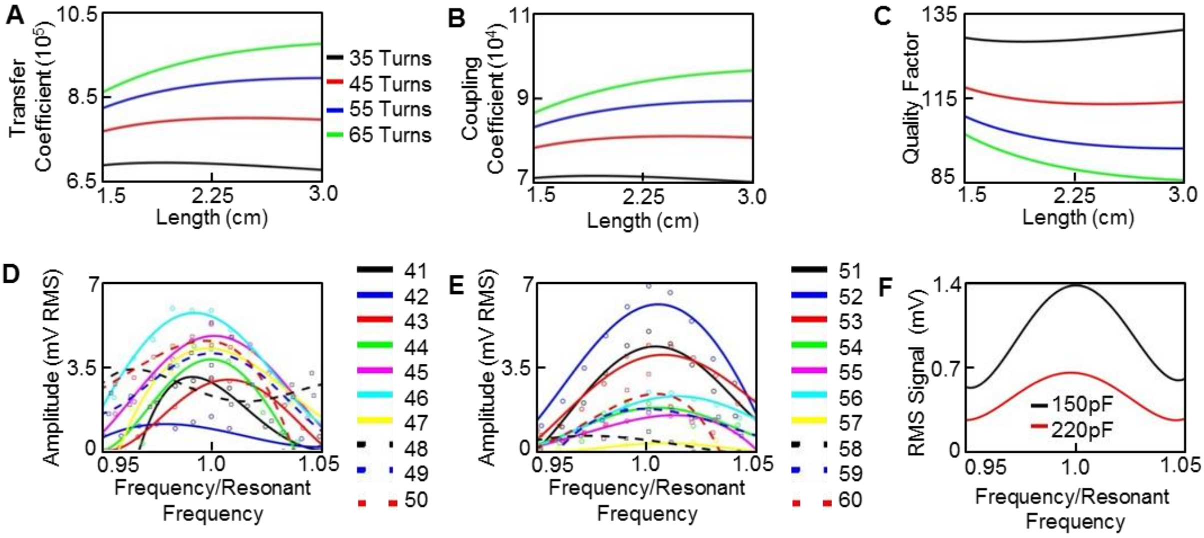

Figure S9. Analytical and experimental optimization of sensor coil for the wireless readout method. A) Transfer coefficient, a measure of power efficiency, and B) coupling coefficient of sensor coil. C) Analytical quality factor of sensor coil. D) Experimental results of received RMS response when varying number of turns of sensor coil from 41 to 50 and E) 51 to 60. F) Increased magnitude of resonance response with lower capacitance and increased quality factor.

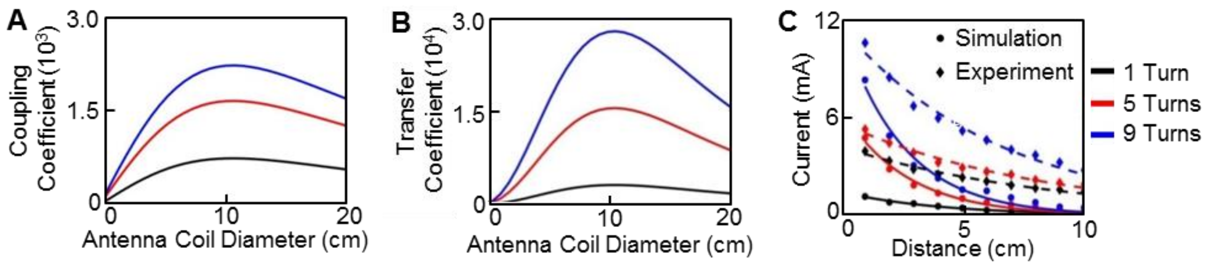

Figure S10. Analytical and experimental optimization of external coils for wireless readout method. A) Coupling coefficient between sensor coil and antenna coils. B) Transfer coefficient of antenna coil. C) Computational and experimental results indicating more excitation coil turns increases induced current.

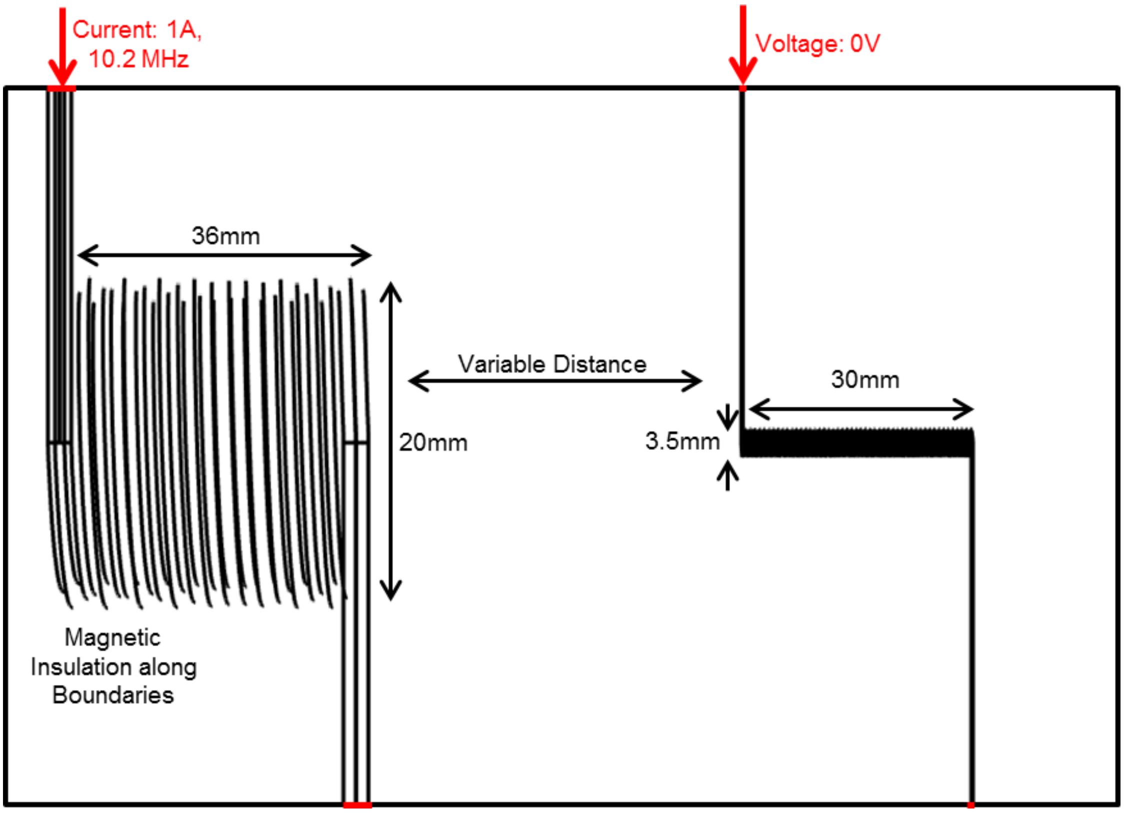

Figure S11. Boundary conditions for 3D computational model. Transmitting coil was excited with a current of 1A, alternating at 10.2 MHz. The sensor coil was not excited with a boundary condition, allowing the transmitting coil to induce current.

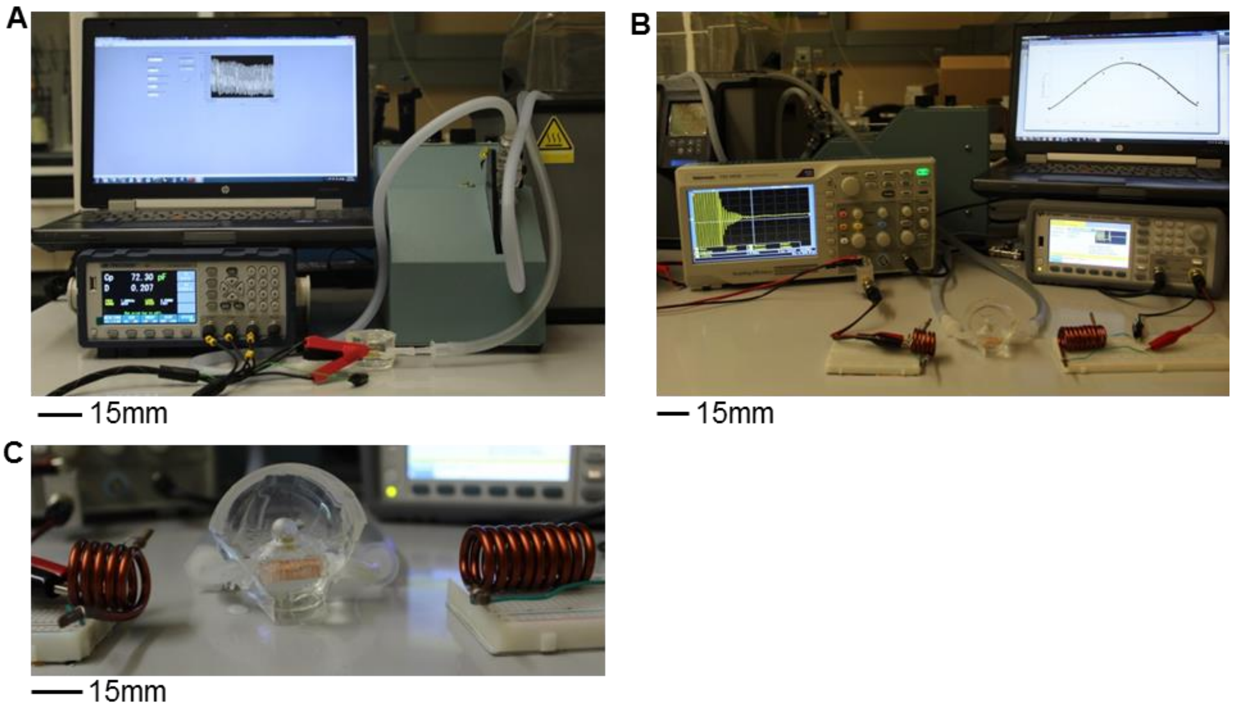

Figure S12. Experimental setup to measure pulsatile flow. A) Measuring flow with LCR meter. B) Wireless system to measure flow. C) Antennas and flow sensor coil for wireless monitoring.

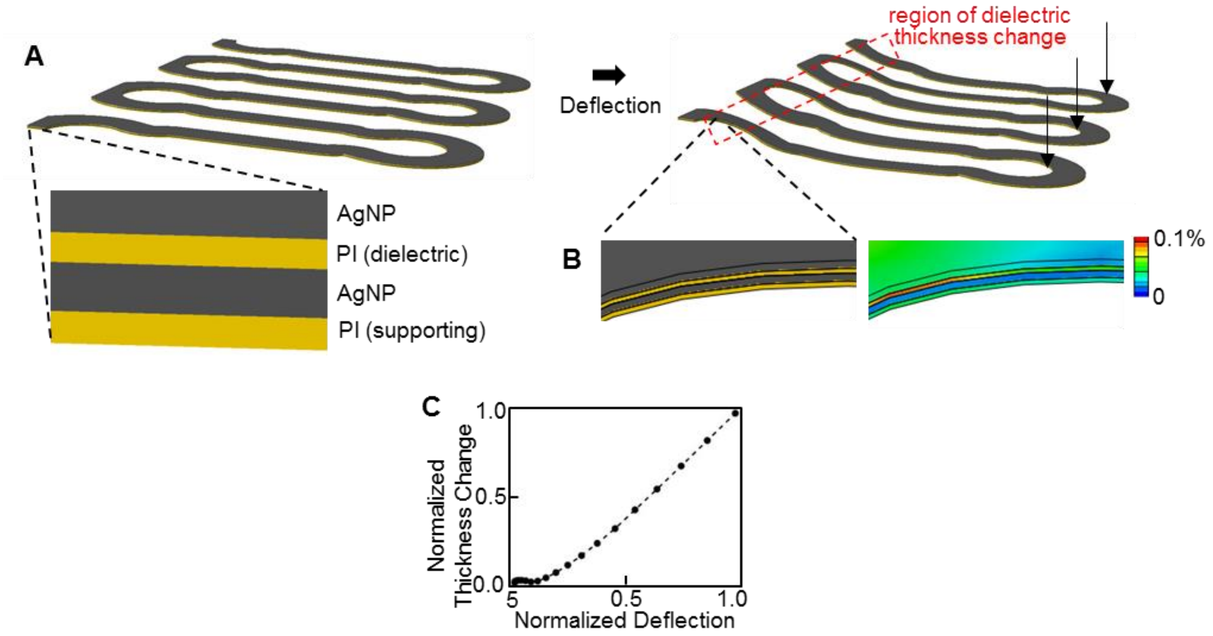

Figure S13. Finite element analysis of mechanism for capacitance change. A) Model geometry with the four sensor layers. A compressive load simulates the effect of flow and deflects the sensor. B) The highest strain occurs in the dielectric layer in the region of bending, indicating the dielectric layer is compressed and causing a change in capacitance. C) The amplitude of deflection correlates with the average dielectric thickness change.

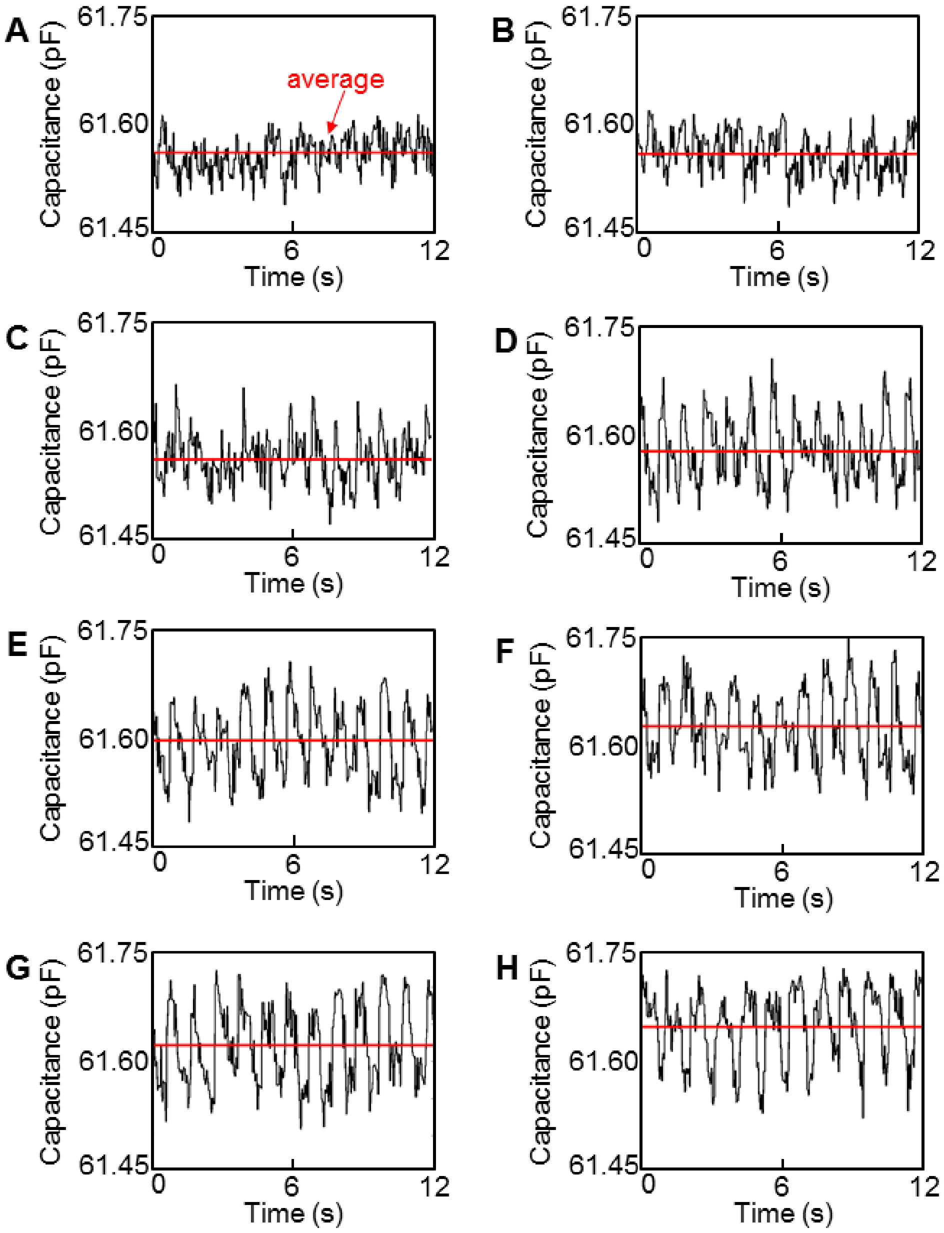

Figure S14. Wired flow measurements according to A-H) 0, 0.05, 0.1, 0.15, 0.2, 0.25, 0.3, and 0.35 ms^{-1}, respectively. The pulsatile flow increases average capacitance.

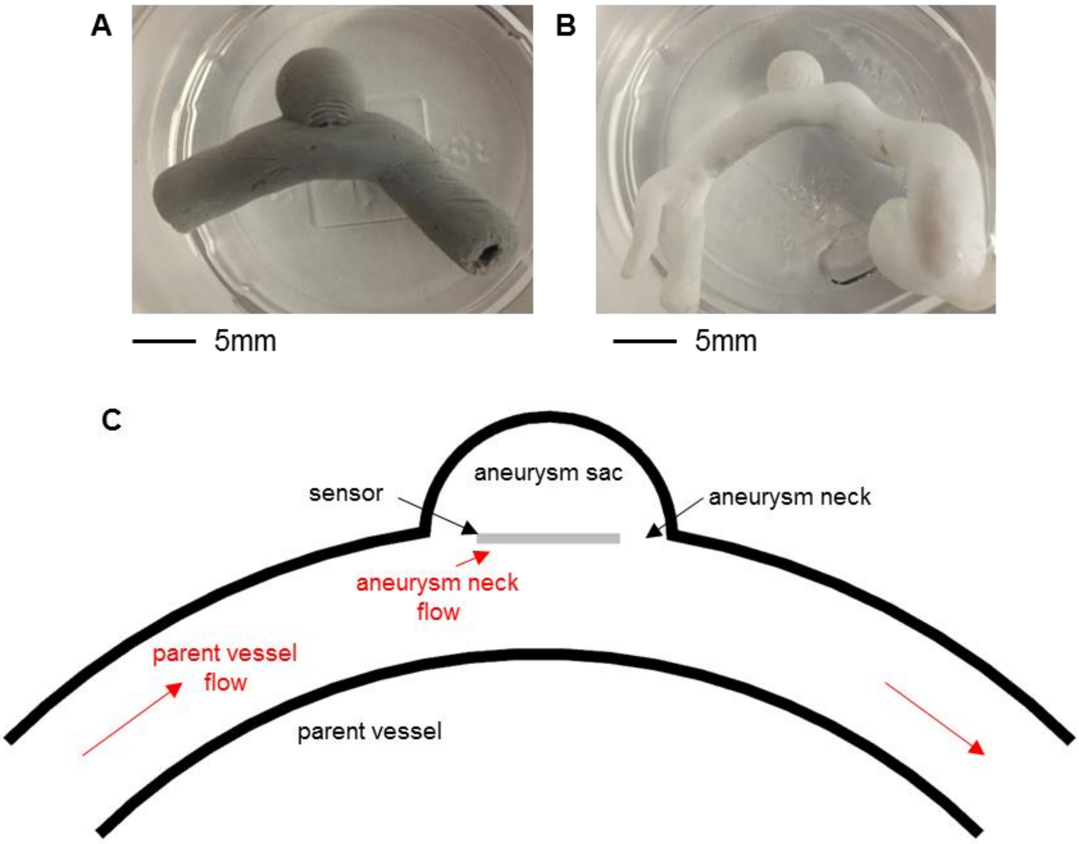

Figure S15. Photograph of PDMS model with 3D printed neurovascular vessels. A) Vessel with 5 mm diameter and aneurysm neck width of 4 mm. B) Highly contoured vessel with 3.5 mm diameter and aneurysm neck width of 2 mm. C) Illustration indicates difference between parent vessel flow and aneurysm neck flow. The sensor is designed to monitor changes in aneurysm neck flow.

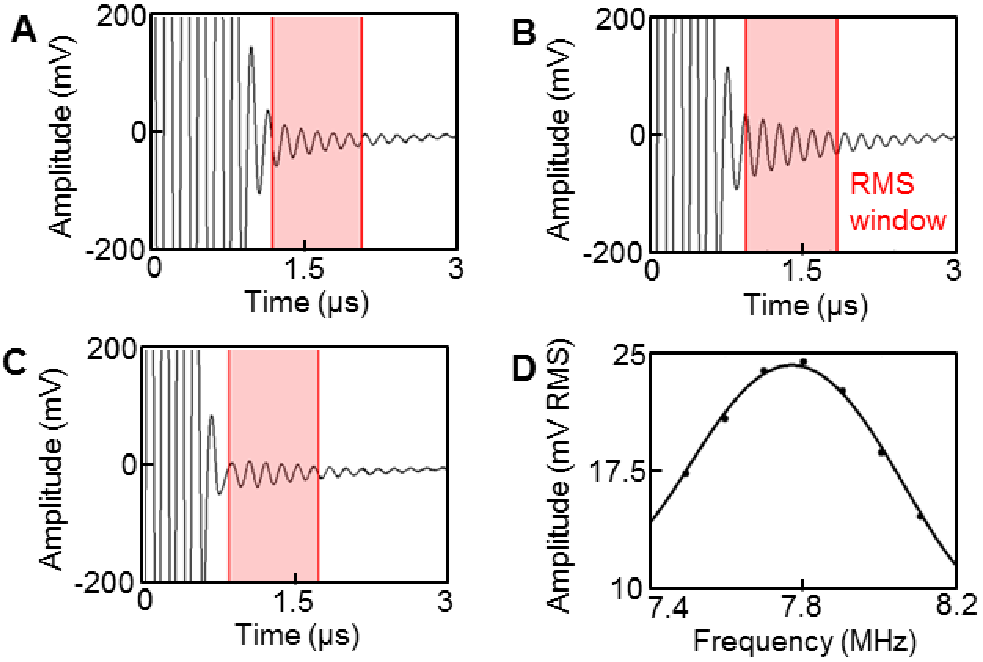

Figure S16. Example frequency sweep for a sensor. Recorded signals at A) 7.4 MHz, B) 7.8 MHz, and C) 8.2 MHz.

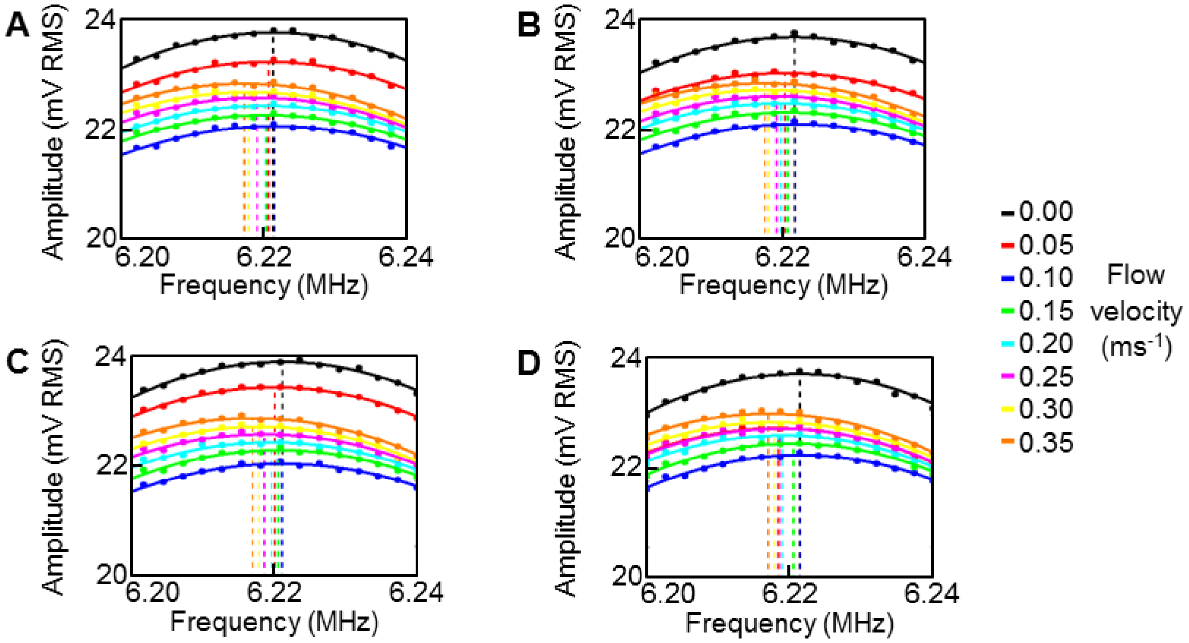

Figure S17. A-D) Additional frequency sweeps used to wirelessly monitor flow.

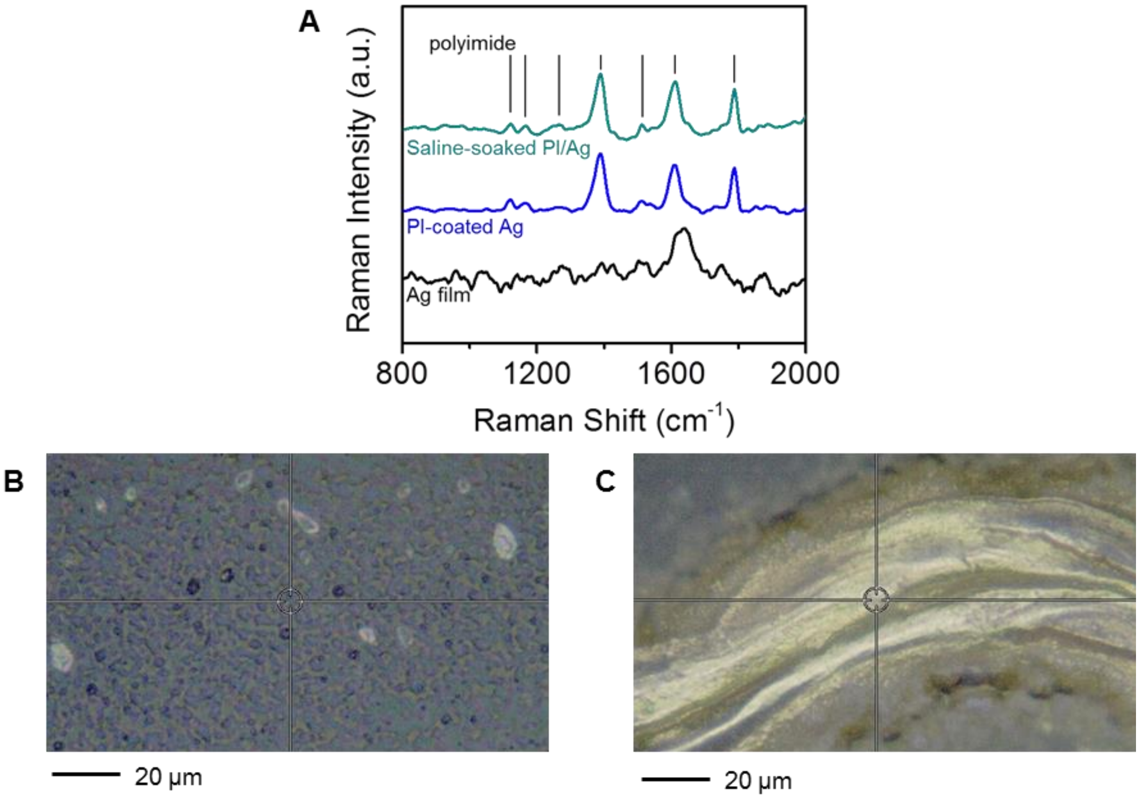

Figure S18. Raman spectroscopy of sintered AgNP film and PI-coated AgNP. A) Raman analysis indicates the AgNP film is fully encapsulated. B) Sintered AgNP film. C) PI coating fully encapsulates the AgNP film.

Move S1. Video of aerosol jet printing of PI for the dielectric layer.

Movie S2. Video of aerosol jet printing of AgNPs as the top electrode in a sensor.

Movie S3. Video of sensor deflection at aneurysm neck during pulsatile flow.
